# Ischemia reperfusion injury provokes adverse left ventricular remodeling in dysferlin-deficient hearts through a pathway that involves TIRAP dependent signaling

**DOI:** 10.1038/s41598-020-71079-7

**Published:** 2020-08-24

**Authors:** Sarah Evans, Carla J. Weinheimer, Attila Kovacs, Jesse W. Williams, Gwendalyn J. Randolph, Wenlong Jiang, Philip M. Barger, Douglas L. Mann

**Affiliations:** 1 Center for Cardiovascular Research, Cardiovascular Division, Division of Cardiology, Washington University School of Medicine, 660 S. Euclid Ave,, Campus Box 8086, St. Louis, MO 63110 USA; 2 Department of Pathology and Immunology, Washington University School of Medicine, St. Louis, MO USA

**Keywords:** Cardiology, Cardiovascular biology, Inflammation, Membrane trafficking

## Abstract

Cardiac myocytes have multiple cell autonomous mechanisms that facilitate stabilization and repair of damaged sarcolemmal membranes following myocardial injury. Dysferlin is a protein which facilitates membrane repair by promoting membrane resealing. Although prior studies have shown that dysferlin-deficient (*Dysf*^*−/−*^) mouse hearts have an impaired recovery from acute ischemia/reperfusion (I/R) injury ex vivo, the role of dysferlin in mediating the recovery from myocardial injury in vivo is unknown. Here we show that *Dysf*^*−/−*^ mice develop adverse LV remodeling following I/R injury secondary to the collateral damage from sustained myocardial inflammation within the infarct zone. Backcrossing *Dysf*^*−/−*^ mice with mice lacking signaling through the Toll-Interleukin 1 Receptor Domain-Containing Adaptor Protein (*Tirap*^*−/−*^), attenuated inflammation and abrogated adverse LV remodeling following I/R injury. Subsequent studies using Poloxamer 188 (P188), a membrane resealing reagent, demonstrated that P188 did not attenuate inflammation nor prevent adverse LV remodeling in *Dysf*^*−/−*^ mice following I/R injury. Viewed together these studies reveal a previously unappreciated role for the importance of membrane sealing and the resolution of inflammation following myocardial injury.

## Introduction

The sarcolemmal phospholipid bilayer membrane that envelopes cardiac myocytes is essential for cell viability by providing a structural barrier function that separates the cytosolic components of the cell from the extracellular environment, as well as a crucial functional role by integrating important cellular functions, such as excitation contraction coupling. Given the importance of the sarcolemma to cell viability, it is not surprising that cardiac myocytes have multiple cell autonomous mechanisms that facilitate stabilization and repair of damaged sarcolemmal membranes. These include sarcolemma phospholipid rearrangements at the site of very small disruptions/injury in order to promote membrane resealing, as well as several endogenous mechanisms that are activated in order to repair damage to preserve muscle cell integrity and viability (reviewed in^1,2^).

In myocardial ischemia–reperfusion (I/R) injury the integrity of the cardiac sarcolemma is severely stressed during ischemia and reperfusion, which leads to membrane tears, blebbing and rupture, and directly contributes to cardiac myocyte dysfunction and cardiac myocyte cell death^3^. The loss of sarcolemmal barrier function can also lead to additional myocardial damage days to weeks following reperfusion secondary to the brisk inflammatory response that ensues following the release of damage associated molecular patterns [DAMPs] by dying cells. DAMPs released by necrotic cardiac myocytes are sufficient to provoke a brisk inflammatory response in the heart that requires intact signaling through Toll-like receptors (TLR4) that are present on the cell surface of cardiac myocytes and cardiac resident immune cells^4,5^.

We have shown previously that tumor necrosis factor (TNF), a pro-inflammatory cytokine released by cardiac myocytes and immune cells in response to ischemic tissue injury, confers cytoprotective responses in the heart that are mediated, at least in part, through the upregulation of an emergency response gene termed dysferlin^6–8^, that has been implicated in mediating rapid membrane repair through its ability to direct intracellular vesicles to sites of membrane injury^9^. Prior studies from this and other laboratories have shown that hearts from dysferlin-deficient mice have an impaired ability to recover from acute ischemia/reperfusion (I/R) injury ex vivo^8,10^. However, the role of dysferlin in chronic models of myocardial injury, wherein the heart is exposed to the dual mechanical stresses imposed by left ventricular (LV) remodeling and activation of the sympathetic nervous system, as well as the stress imposed by activation of the innate immune response, is not known. Here we show that mice lacking dysferlin (*Dysf*^*−/−*^ mice) undergo adverse LV cardiac remodeling following I/R injury secondary to increased myocardial inflammation that is mediated by Toll-Interleukin 1 Receptor (TIR) Domain-Containing Adaptor Protein (TIRAP) signaling in the heart.

## Results

### Ischemia–reperfusion injury provokes adverse LV remodeling in dysferlin deficient mice through a TIRAP dependent pathway

**Figure 1:**
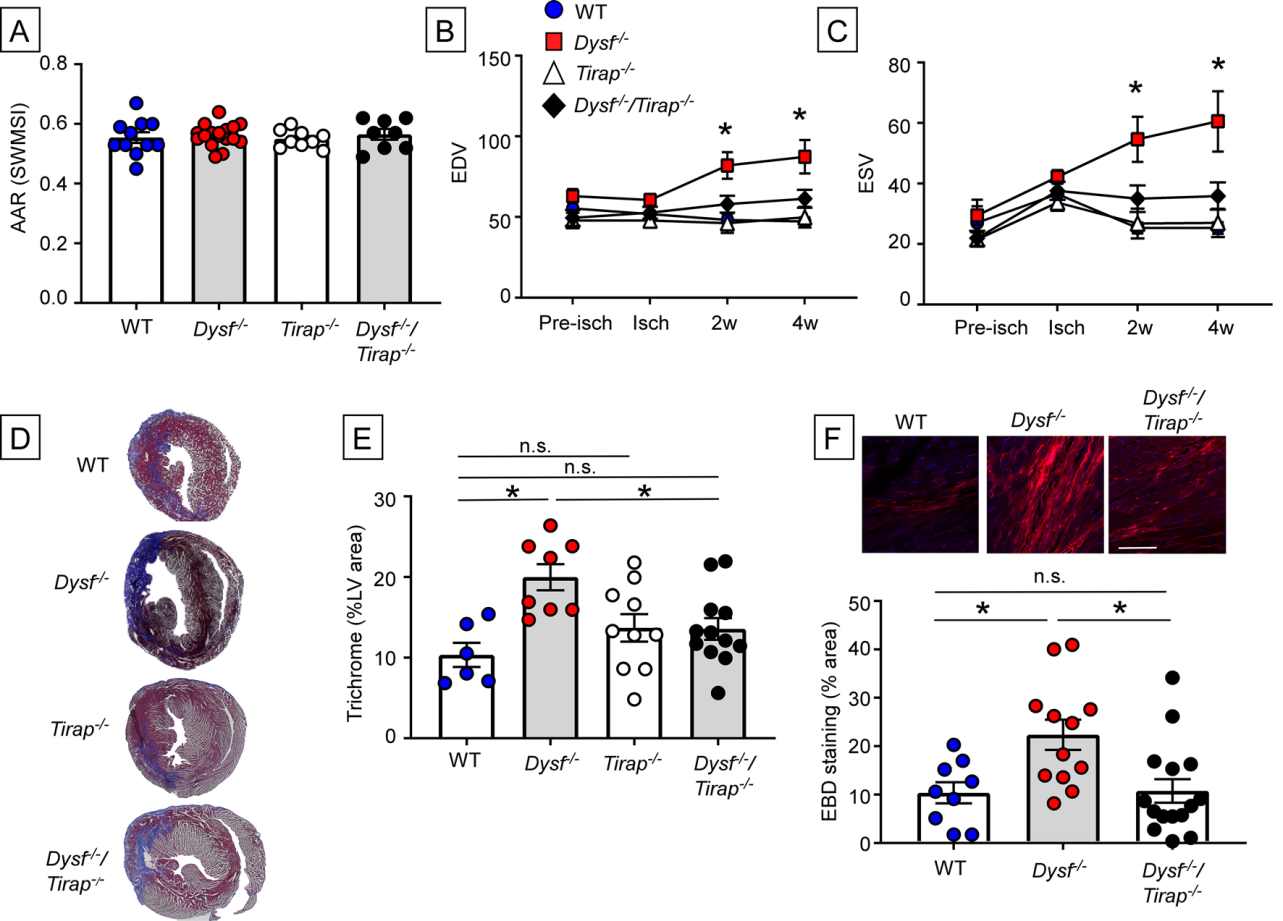
Effect of ischemia reperfusion (I/R) injury in WT*, Dysf*^*−/−*^, *Tirap*^*−/−*^ and *Dysf*^*−/−*^/*Tirap*^*−/−*^ mouse hearts. WT (n = 11), *Dysf*^*−/−*^ (n = 17), *Tirap*^*−/−*^ (n = 9), *Dysf*^*−/−*^/*Tirap*^*−/−*^ (n = 8) mice underwent closed chest ischemia (60 min) followed by 2 to 4 weeks of reperfusion. Mice were imaged by 2-D echocardiography at baseline (pre-ischemia), during the imposition of ischemia, and at 2 and 4 weeks. (**A**) Area-at-risk (AAR(SWMSI)), (**B**) Left ventricular end-diastolic volume (EDV), (**C**) Left ventricular end systolic volume (ESV) (*p < 0.05 compared to WT), (**D**) Representative images of trichrome staining of histological myocardial sections. 2 weeks after reperfusion, (**E**) Group data for trichrome staining (expressed as a % of the LV myocardium) 2 weeks after reperfusion (n = 6–12 per group), (**F**) Representative images and group data for Evans Blue dye (EBD) uptake (expressed as a % of the LV myocardium) 2 weeks after reperfusion (n = 9–12 images/group). Scale bar = 100 µm. Data are presented as mean ± SEM (*p < 0.05).

We previously identified dysferlin as a cytoprotective protein that mediates the beneficial effects of TNF/TRAF2 signaling in Langendorff buffer perfused hearts subjected to no-flow ischemia reperfusion injury^8^. To extend these studies in vivo, wherein the extent of tissue injury can be modulated by the autonomic nervous system and the immune system, we performed closed-chest ischemia reperfusion studies in wild-type (WT) and *Dysf*^*−/−*^ mice. Figure 1 shows that LV remodeling was significantly greater in the *Dysf*^*−/−*^ hearts when compared to WT mouse hearts. LV end-diastolic volume (EDV) (Fig. 1B) (p < 0.001 at 2 and 4 weeks of reperfusion), end-systolic volume (ESV) (Fig. 1C) (p = 0.003 at 2 weeks and p = 0.004 at 4 weeks of reperfusion), and LV mass (LVM) (p = 0.008 at 2 weeks, p < 0.001 at 4 weeks) were significantly increased in *Dysf*^*−/−*^ hearts compared to WT (Supplemental Figure S1), whereas the LV ejection fraction (LVEF) was significantly decreased (p < 0.001 at both 2 and 4 weeks) in the *Dysf*^*−/−*^ hearts. Although the area-at-risk (AAR) was not significantly different (p > 0.999) in WT and *Dysf*^*−/−*^ mice, the infarct size by Masson’s trichrome staining was significantly greater (p = 0.003) in *Dysf*^*−/−*^ hearts when compared to WT at 2 weeks (Fig. 1D,E). Consistent with our prior studies ex vivo^8^, the uptake of Evans Blue dye (EBD) was significantly increased (p = 0.02) in the hearts of *Dysf*^*−/−*^ mice when compared to WT hearts (Fig. 1F), suggesting that there was increased cell necrosis.

In order to determine the mechanism(s) for the adverse LV remodeling in the *Dysf*^*−/−*^ mouse hearts, we backcrossed *Dysf*^*−/−*^ mice with mice lacking the Toll-Interleukin 1 Receptor (TIR) Domain-Containing Adaptor Protein (TIRAP), which is involved in bridging the myeloid differentiation primary response gene 88 (MyD88) to the receptor complex for TLR2 and TLR4 signaling in response to bacterial infection or tissue injury, in order to generate *Dysf*^*−/−*^/*Tirap*^*−/−*^ double knock out mice. The rationale for these studies was two-fold: first, we and others have shown the release of damage associated molecular patterns (DAMPs) by necrotic cells leads to increased cardiac inflammation that is mediated, at least in part, through Toll-like receptor 4 (TLR4) signaling pathways^5^; second, a prior study in dysferlin null mice showed that there was less inflammation in mice that lacked MyD88^11^, a canonical adaptor protein that interacts with TIR domain-containing proteins, and which mediates inflammatory signaling pathways through TLR signaling pathways^12^. The salient finding shown in Fig. 1B,C is that there was less adverse LV remodeling in *Dysf*^*−/−*^/*Tirap*^*−/−*^ mice at 2 or 4 weeks of age. Both LV EDV (p = 0.70 at 2 weeks, p = 0.42 at 4 weeks) and ESV (p = 0.70 at 2 weeks, p = 0.78 at 4 weeks) were not significantly different than the values observed in WT hearts. Moreover, LV EF (p = 0.069 at 2 weeks, p = 0.267 at 4 weeks) and LVM (p = 0.746 at 2 weeks, p = 0.678 at 4 weeks) were not significantly different in the *Dysf*^*−/−*^/*Tirap*^*−/−*^ hearts when compared to WT (Supplemental Figure S1). Both AAR and infarct size (Fig. 1A,D,E) were not significantly different in the in *Dysf*^*−/−*^/*Tirap*^*−/−*^ hearts when compared to WT (p > 0.999 and p = 0.724, respectively). Consistent with these observations, the extent of EBD uptake was not significantly different (p > 0.999) in *Dysf*^*−/−*^*/Tirap*^*−/−*^ and WT hearts (Fig. 1F). Viewed together these findings show that the adverse LV remodeling observed in *Dysf*^*−/−*^ mice is TIRAP-dependent, which suggests that the adverse LV remodeling observed in the *Dysf*^*−/−*^ mice was secondary to collateral damage mediated by TIRAP-dependent myocardial inflammation.

### I/R-induced inflammation in dysferlin deficient mice is TIRAP dependent

**Figure 2:**
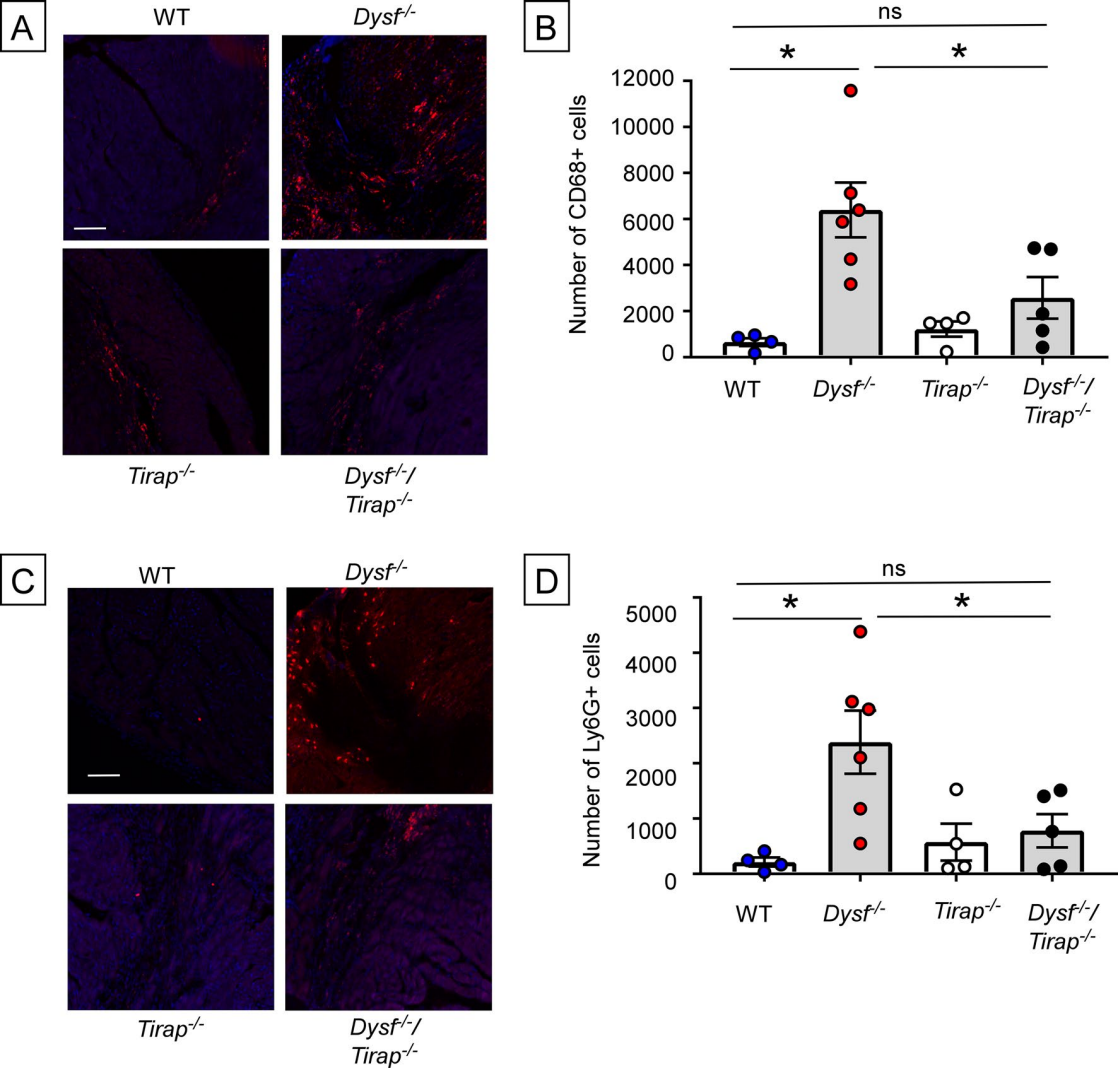
Myocardial inflammation within the infarct zone of WT*, Dysf*^*−/−*^, *Tirap*^*−/−*^ and *Dysf*^*−/−*^/*Tirap*^*−/−*^ mouse hearts following I/R injury. The extent of myocardial inflammation within the infarct zone was determined by enumerating the number of CD68+ and Ly6G+ cells in the hearts of WT, *Dysf*^*−/−*^, *Tirap*^*−/−*^ and *Dysf*^*−/−*^/*Tirap*^*−/−*^ mouse hearts 2 weeks after I/R injury. (**A**) Representative fluorescent images of CD68+ staining, (**B**) Group data for the number of CD68+ cells (n = 4–6 myocardial sections/group), (**C**) Representative fluorescent images of Ly6G+ staining, (**D**) Group data for the number of Ly6G + cells w (n = 4–6 myocardial sections/group). Scale bars = 100 µm, Data are presented as mean ± SEM (*p < 0.05).

Based on prior studies demonstrating that increased membrane fragility in *Dysf*^*−/−*^ mice leads to increased skeletal muscle inflammation^13^ and demonstrating a role for TLR2 in I/R induced inflammation^14^, we hypothesized that membrane fragility contributed to the adverse LV remodeling in *Dysf*^*−/−*^ hearts secondary to increased inflammation that was mediated through TIRAP-dependent signaling pathways that were activated by the release of DAMPs from damaged and/or dying cells. Figure 2A shows representative immunofluorescence staining of CD68+ cells in *Dysf*^*−/−*^ and WT hearts 2 weeks after closed-chest I/R injury; Fig. 2B summarizes the results of group data. As shown, there was a significant (p = 0.002) increase in the number of CD68+ cells within the infarct zone of the *Dysf*^*−/−*^ hearts compared to WT hearts. We also observed a significant (p = 0.01) increase in Ly6G+ cells (Fig. 2C,D) in the infarct zone of the *Dysf*^*−/−*^ hearts when compared to WT hearts. Remarkably, there was no significant increase in CD68+ (p = 0.7) and Ly6G+ (p > 0.999) cells within the infarct zone of *Dysf*^*−/−*^/*Tirap*^*−/−*^ hearts when compared to WT hearts.

### Bone marrow derived dysferlin deficient hematopoietic cells do not contribute to I/R-induced adverse LV remodeling in *Dysf*^*−/−*^ mice

Given that dysferlin influences both the trafficking and function of immune cells^15,16^, we also performed experiments, wherein we reconstituted the bone marrow of *Dysf*^*−/−*^ mice with WT mice donor cells, as well as reciprocal experiments wherein we reconstituted the bone marrow of WT mice with *Dysf*^*−/−*^ donor cells, and then subjected these chimeric mice to I/R injury 8 weeks after they were reconstituted. These studies showed that 2 weeks after I/R injury there was no difference in the AAR, LV EDV, LV ESV, LV EF, nor LV mass in *Dysf*^*−/−*^ → WT chimeric mice when compared to WT → WT chimeric mice (see Supplemental Figure S2 for details). There was, however, a significant increase in LV EDV (p = 0.030) and LV ESV (p = 0.041) in WT → *Dysf*^*−/−*^ mice , suggesting that bone marrow derived dysferlin deficient cells do not contribute to adverse LV remodeling after I/R injury in *Dysf*^*−/−*^ mice.

### Effect of Poloxamer 188 on I/R-induced adverse LV remodeling in dysferlin deficient mice

The findings that the increase in myocardial inflammation and adverse LV remodeling observed in *Dysf*^*−/−*^ mouse hearts was abrogated in *Dysf*^*−/−*^/*Tirap*^*−/−*^ mouse hearts, but not in chimeric *Tirap*^*−/−*^* → Dysf*^*−/−*^ mouse hearts suggested that the adverse phenotype in the *Dysf*^*−/−*^ mouse hearts was cardiac autonomous. Noting that prior studies demonstrated that dysferlin plays an important role in membrane sealing^17,18^, and that the membrane resealing reagent Poloxamer 188 (P188) was cardioprotective in *Dysf*^*−/−*^ mouse hearts subjected to I/R injury ex vivo^2,10^, we sought to determine whether treatment with P188 would rescue the phenotype of the *Dysf*^*−/−*^ mice subjected to I/R injury in vivo. We first confirmed that in our hands P188 (10 µM) improved LV functional recovery (p = 0.007) in *Dysf*^*−/−*^ mouse hearts subjected to 30 min of no-flow ischemia, followed by 60 min of reperfusion ex vivo (Supplemental Figure S3), as suggested by Martindale et al.^10^. Interestingly, recovery of LV function following I/R injury ex vivo was not significantly different (p = 0.826) in WT hearts treated with P188 when compared to diluent treated hearts. As we have reported previously^8^, there were small but statistically significant differences in LV developed pressure between WT (Figure S3A) and *Dysf*^*−/−*^ (Figure S3B) mouse hearts following I/R injury (p = 0.035 by ANOVA).

**Figure 3:**
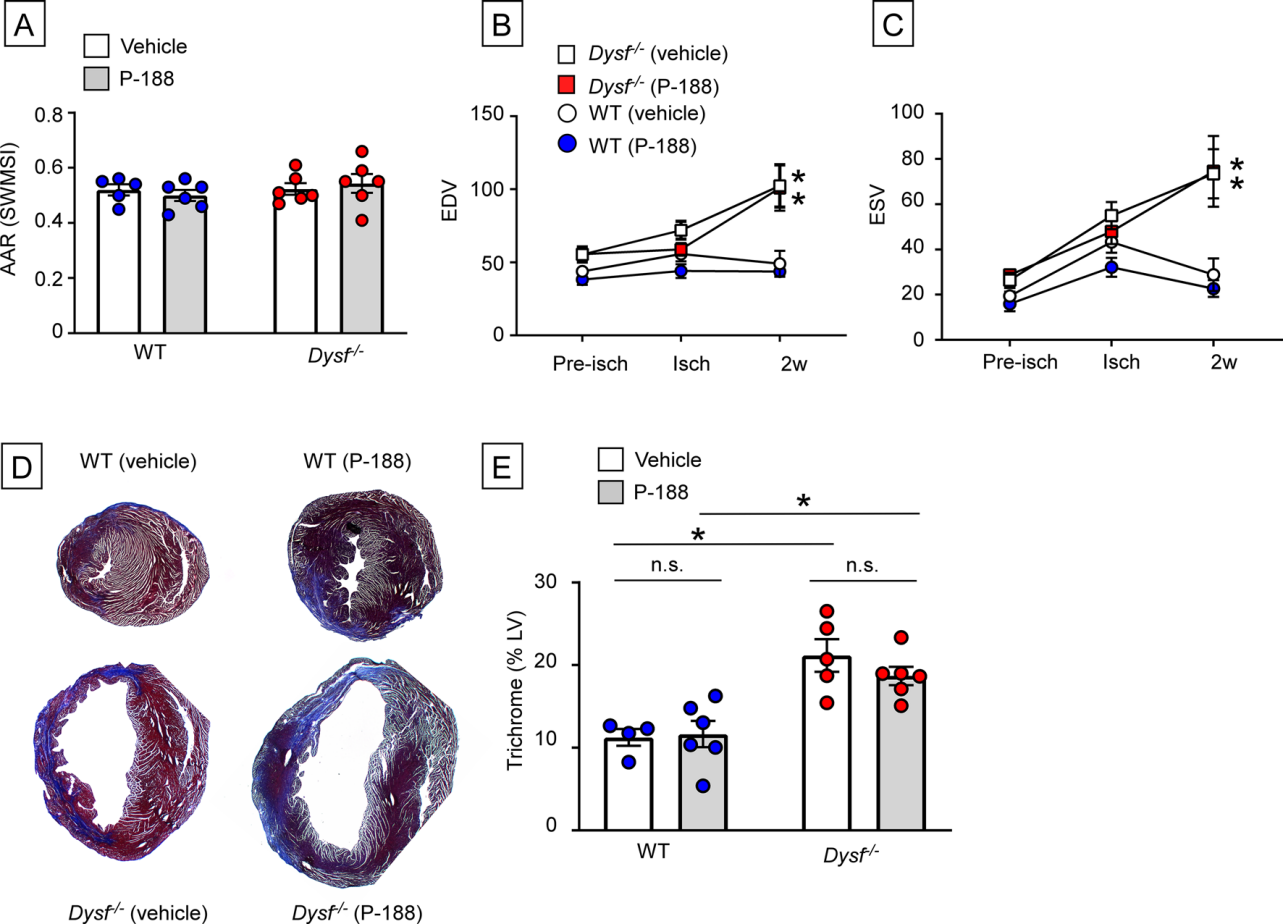
Effect of Poloxamer 188 (P188) on LV remodeling and infarct size in WT and *Dysf*^*−/−*^ mice following I/R injury. WT and *Dysf*^*−/−*^ mice, subjected to closed chest ischemia (60 min) or a sham procedure followed by 2 weeks of reperfusion, were treated with P188 or an equal volume of diluent immediately prior to reperfusion and then daily for 14 days. Mice were imaged by 2-D echocardiography at baseline (pre-ischemia), during the imposition of ischemia, and at 2 after reperfusion (n = 5–6/group). (**A**) Area-at-risk (AAR(SWMSI)), (**B**) Left ventricular end-diastolic volume (EDV), (**C**) Left ventricular end-systolic volume (ESV) (**D**) Representative images of trichrome staining of histological myocardial sections. 2 weeks after reperfusion, (**E**) Group data for trichrome staining (expressed as a % of the LV myocardium) 2 weeks after reperfusion (n = 4–6 per group). Data are presented as mean ± SEM (*p < 0.05 compared to WT).

After confirming that P188 was cardioprotective in *Dysf*^*−/−*^ mice ex vivo, we treated WT and *Dysf*^*−/−*^ mice with P188 in vivo immediately after closed chest I/R injury. For these studies P188 (or diluent) was administered via the jugular vein immediately after reperfusion, followed by an i.p. injection of diluent or P188 6 h later. Subsequently, the mice were given daily i.p injections of diluent or P188 up until the time of terminal sacrifice at 14 days. Figure 3A shows that the AAR risk was not significantly different in the WT (p > 0.999) and *Dysf*^*−/−*^ (p > 0.999) hearts treated with either diluent or P188. Consistent with our earlier studies (Fig. 1), we observed a significant increase in LV remodeling in diluent treated *Dysf*^*−/−*^ mice when compared to diluent treated WT mice (LV EDV p = 0.0001; LV ESV p = 0.0003; Fig. 3). As shown in Fig. 3B,C, I/R-induced LV remodeling was not significantly different in the WT mice treated with P188 or diluent (LV EDV p > 0.999; LV ESV p > 0.999). However, the salient finding shown by Fig. 3 and Supplemental Figure 4 is that treatment with P188 did not attenuate LV remodeling in the *Dysf*^*−/−*^mice, when compared to diluent treated *Dysf*^*−/−*^ mice (LV EDV p > 0.999; LV ESV p > 0.999). Infarct size was not significantly different in the P188 treated WT mice (p > 0.999) or the P188 treated *Dysf*^*−/−*^mice (p > 0.999), relative to diluent treated WT and *Dysf*^*−/−*^ mice.

**Figure 4:**
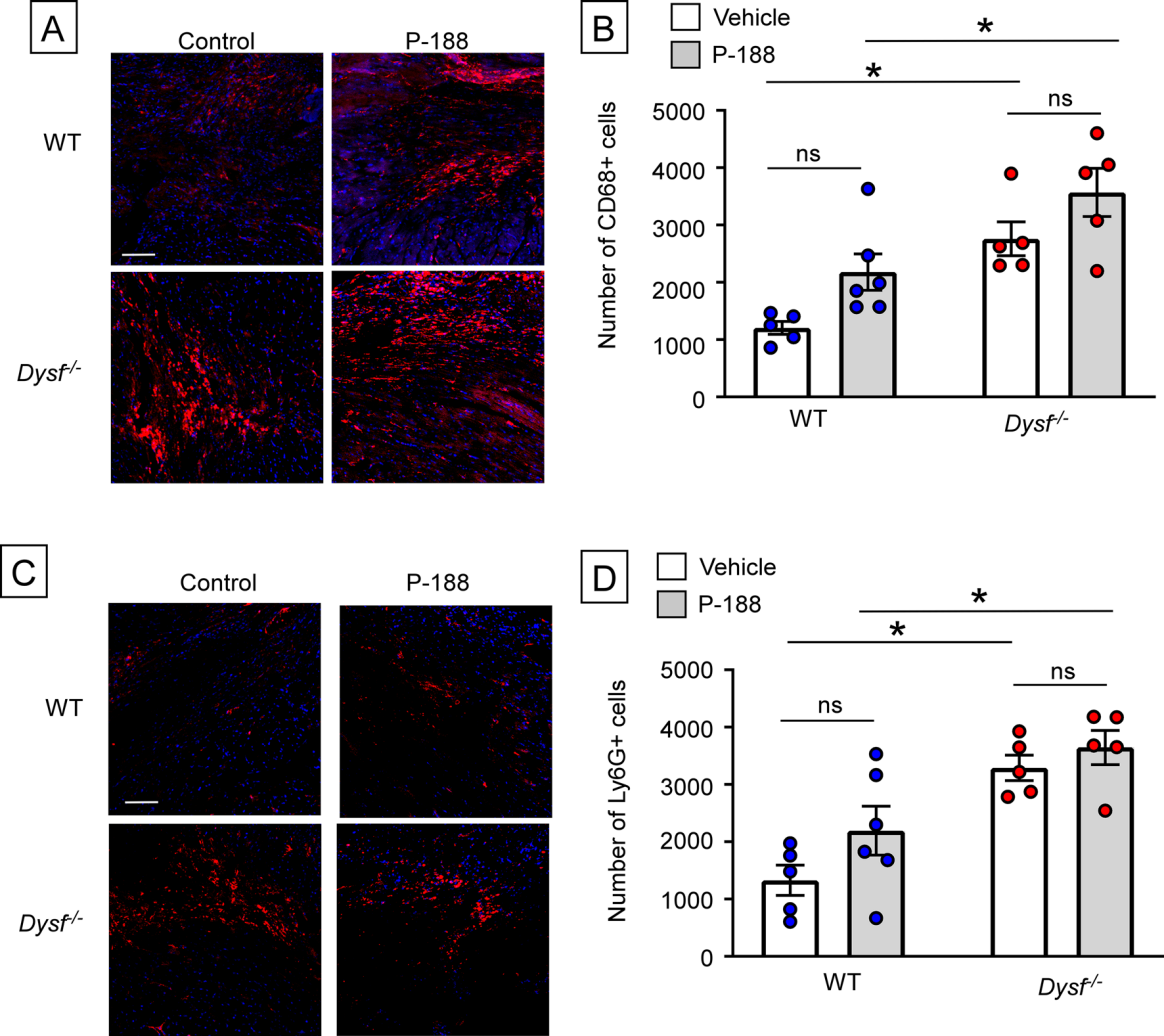
Effect of Poloxamer 188 (P188) on myocardial inflammation within the infarct zone in WT and *Dysf*^*−/−*^ mice following I/R injury. The extent of myocardial inflammation within the infarct zone was determined by enumerating the number of CD68+ and Ly6G+ cells in the hearts of diluent and P188 treated WT and *Dysf*^*−/−*^ mice 2 weeks following sham procedure or closed chest I/R injury. (**A**) Representative fluorescent images of CD68+ staining within the infarct zone, (**B**) Group data for the number of CD68+ cells within the infarct zone (n = 5–6 myocardial sections/group), (**C**) Representative fluorescent images of Ly6G+ staining within the infarct zone, (**D**) Group data for the number of Ly6G+ cells within the infarct zone 2 weeks following reperfusion (n = 5–6 myocardial sections/group). Scale bars = 100 µm. Data are presented as mean ± SEM (*p < 0.05).

To determine whether P188 had an effect on I/R-induced inflammation, we quantified the number of CD68+ and Ly6G+ cells in diluent and P188 treated WT and *Dysf*^*−/−*^ mouse hearts 2 weeks after I/R injury. Figure 4A shows representative immunofluorescence staining of CD68+ cells in diluent treated and P188 treated *Dysf*^*−/−*^ and WT hearts, whereas Fig. 4B summarizes the results of group data. As shown, there was a significant increase in the number of CD68+ cells in the diluent (p = 0.012) and P188 (p = 0.020) treated *Dysf*^*−/−*^ hearts when compared to diluent and P188 treated WT mouse hearts. Figure 4C shows representative immunofluorescence staining of Ly6G+ cells in diluent treated and P188 treated *Dysf*^*−/−*^ and WT hearts 2 weeks after closed-chest I/R injury, and Fig. 4D summarizes the results of group data. There was a significant increase (p = 0.003) in the number of Ly6G+ cells in the diluent treated *Dysf*^*−/−*^ hearts when compared to diluent treated WT mouse hearts. There was also a significant increase (p = 0.022) in the number of Ly6G+ cells in the P188 treated *Dysf*^*−/−*^ hearts when compared to P188 treated WT mouse hearts. Although there was a numerical increase in the number of CD68+ and Ly6G+ cells in the P188 treated WT hearts when compared to diluent treated hearts, these changes were not statistically significant (p = 0.149 and 0.298, respectively). There was no significant difference in the number of CD68+ (p = 0.365) and Ly6G+ (p > 0.999) cells in the diluent and P188 treated *Dysf*^*−/−*^ hearts.

**Figure 5:**
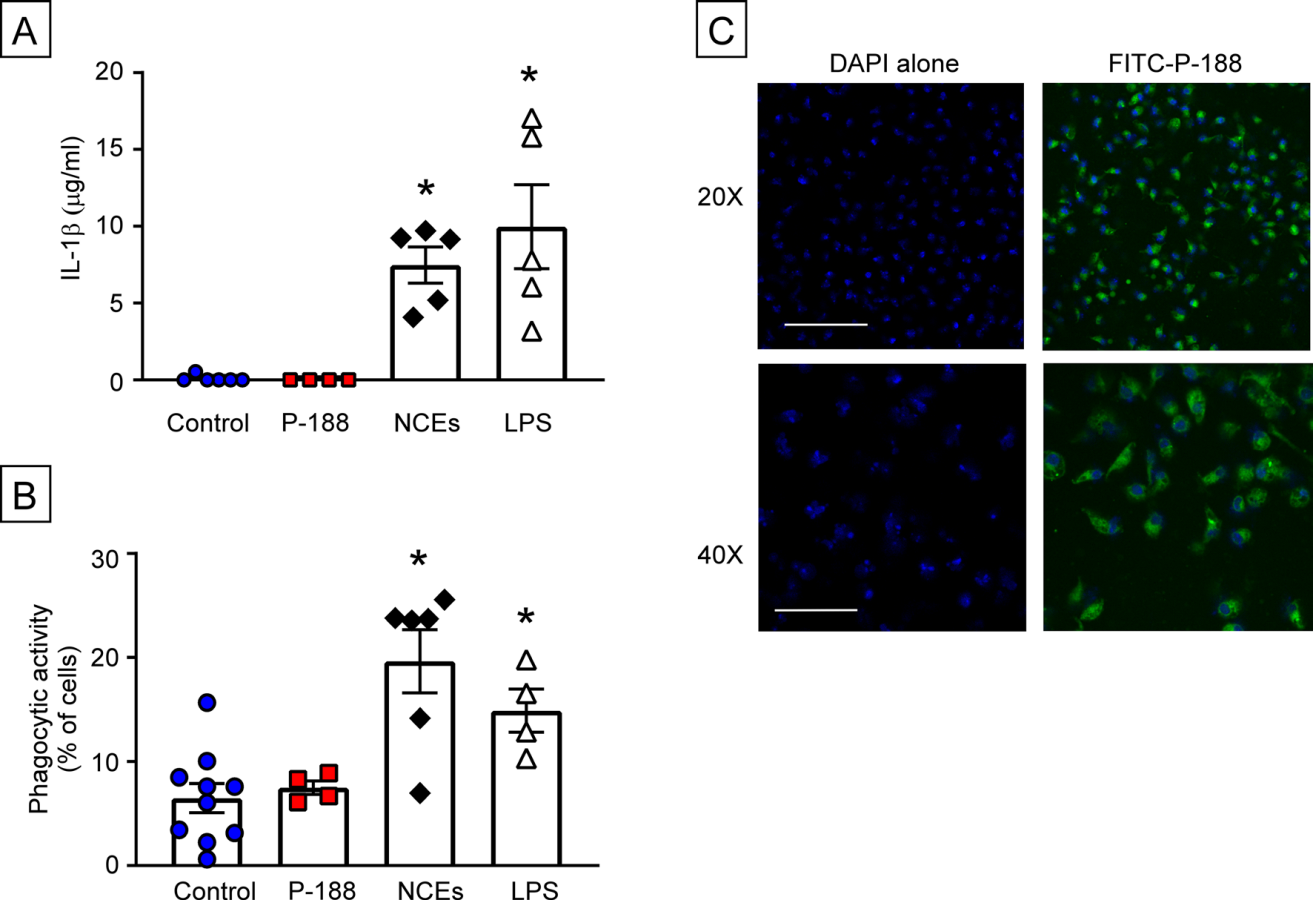
Effect of Poloxamer 188 (P188) on isolated peritoneal macrophages (ipMACs). To determine whether P188 had intrinsic effects on isolated peritoneal macrophages, we stimulated cultures of ipMACs for 24 h with diluent (control), necrotic myocardial cell extracts (NCEs) or LPS (positive controls), as well as P188. (**A**) IL-1β levels in supernatants of ipMAC cultures (n = 4–6 samples/group), (**B**) Bead uptake by cultured ipMACs (n = 4–10 samples per group), (**C**) Representative fluorescence images of FITC-labeled P188 uptake by ipMACs following 18 h of incubation. DAPI staining (blue) was used to identify the nuclei of ipMACs. Scale bar ×20 = 100 µm. Scale bar ×40 = 50 µm. Data are presented as mean ± SEM (*p < 0.05 compared to control).

As a first step towards understanding why P188 was cytoprotective ex vivo, but had no effect in vivo, we performed studies with P188 and cultured intraperitoneal macrophages (ipMACs). Based on a prior study which showed that P188 activates immune cells^19^, we asked whether P188 provoked increased IL-1β secretion and increased phagocytosis in ipMACs. Figure 5A shows that stimulation with P188 did not increase (p > 0.999) IL-1 β secretion when compared to diluent treated control ipMACs, whereas treatment with necrotic myocardial cell extracts (NCEs) and LPS (positive control) provoked a significant (p = 0.006 and p = 0.0005 respectively) increase in IL-1β secretion relative to diluent treated controls. Figure 5B, shows that relative to diluent treated cells, treatment with P188 did not lead to increased phagocytic activity (p > 0.999), whereas stimulation with necrotic myocardial extracts (NCEs) (p = 0.0002) and LPS (p = 0.0322) increased phagocytosis significantly. Lastly, as shown in Fig. 5C, we observed that FITC- labeled P188 was internalized by ipMACs (18 h). Viewed together these data suggest that although P188 is phagocytosed by macrophages, it has no intrinsic effect of macrophage pro-inflammatory or phagocytic activities.

## Discussion

The phospholipid bilayer sarcolemmal membrane that envelops cardiac myocytes presents the first line of defense for preserving cell viability following myocardial injury. Here we show that dysferlin null mice, which lack an effective membrane repair mechanism are more sensitive to I/R than WT mice, and undergo adverse LV remodeling secondary to sustained TIRAP-dependent inflammation within the infarct zone, resulting in increased myocyte cell death and increased infarct size. The following lines of evidence support this statement. First, I/R injury resulted in a significant increase in adverse cardiac remodeling in *Dysf*^*−/−*^ mice, when compared to WT controls (Fig. 1B). Although the area at risk was not significantly different in the WT and *Dysf*^*−/−*^ mice (Fig. 1A), the infarct size at 2 weeks was significantly greater in the *Dysf*^*−/−*^ mouse hearts (Fig. 1E). Consistent with the increase in infarct size, we observed increased uptake of EBD in the *Dysf*^*−/−*^ cardiac myocytes (Fig. 1F), suggesting that there was increased necrotic myocyte cell death in I/R injured *Dysf*^*−/−*^ mouse hearts. Second, crossing the *Dysf*^*−/−*^ mice with *Tirap*^*−/−*^ mice resulted in decreased EBD uptake (Fig. 1F), decreased inflammation in the infarct zone (Fig. 2B,D), smaller infarct sizes (Fig. 1E), and less LV remodeling (Fig. 1B), when compared to *Dysf*^*−/−*^ mice. The adverse LV remodeling in the *Dysf*^*−/−*^ mice was not affected by reconstituting the bone marrow of the *Dysf*^*−/−*^ mice with WT marrow (Supplemental Figure S2), demonstrating that the increased inflammation and adverse LV remodeling in *Dysf*^*–/–*^ mice is mediated centrally and does not require *Dysf*^*–/-*^ bone marrow derived hematopoietic cells.

The observation that the increased inflammation within the infarct zone of *Dysf*^*−/−*^ hearts was abrogated in a TIRAP-deficient background (Fig. 2), suggests (but does not prove) that the inability to reseal membranes in injured *Dysf*^*–/-*^ mouse hearts results in the ongoing release of intracellular molecules (e.g. DAMPs) that engage TLR2/TLR4 receptors on cardiac myocytes and resident immune cells within the infarct zone, leading to sustained inflammation. Similar findings have been observed in skeletal muscle of *Dysf*^*−/−*^ mice^13,20^. Third, given that increased membrane permeability has been proposed as one of the mechanisms for increased tissue injury and inflammation in the *Dysf*^*−/−*^ mice, we treated the *Dysf*^*−/−*^ mice with P188, a non-ionic tri-block copolymer that has been shown to seal membranes, at the time of reperfusion. As shown in Figs. 3 and 4, although treatment with P188 prevented cardiac injury ex vivo, consistent with a prior report^10^, treatment with P188 did not decrease myocardial inflammation, nor attenuate adverse cardiac remodeling following I/R injury in vivo.

### Membrane sealing and myocardial injury

Sarcolemmal integrity is preserved through several cardiac myocyte cell autonomous mechanisms that either stabilize the membrane through phospholipid rearrangements at the site of tissue injury, or directly promote membrane resealing. Thus far, studies have shown that a number of different molecules play an integral role in maintaining membrane integrity including dysferlin, the dystrophin glycoprotein complex (DGC), Mitsugumin 53 (TRIM72), thrombospondin 4, synaptogamins, SNARE proteins, the endosomal sorting complex required for transport and GRAF1^1,2,21–23^. Dysferlin is a type II transmembrane protein belonging to the ferlin family of proteins which have been shown to play an important role in membrane repair by facilitating Ca^2+^-mediated trafficking of vesicles to the site of membrane injury^9,24^. While the absence of dysferlin is known to lead to skeletal muscle damage in limb girdle muscular dystrophy type 2B and Miyoshi myopathy, the effects of dysferlin in the heart are less well known. Two prior studies have shown that *Dysf*^*−/−*^ mice have impaired ability to recover LV function following I/R injury ex vivo^10,25^. A prior in vivo study by Han et al.^17^ demonstrated that infarct size and LV ejection fraction were not significantly different in WT and *Dysf*^*−/−*^ mice following acute coronary artery ligation. Our results significantly expand upon these acute studies by demonstrating that the longer term sequelae of ischemic myocardial injury in *Dysf*^*−/−*^ mice results in significant infarct expansion secondary to TIRAP-dependent increased inflammation within the infarct zone. Our findings in the heart are consistent with prior studies that showed that skeletal muscle performance was improved in dysferlin-deficient mice when crossed with Myd88-deficient mice (TIRAP interacts directly with MyD88)^11,20^. Uaesoontrachoon and colleagues hypothesized that breaches in the skeletal muscle membrane in dysferlin-deficient mice resulted in the release of endogenous danger signals that bind to cognate TLR ligands on muscle and immune cells, which in turn activate downstream processes that lead to the recruitment of inflammatory cells that worsen the initial damage caused by the dysferlin gene defect^11^.

Several studies have used chemical-based membrane stabilizers, such as P188, to protect striated muscle membranes^2,10,26–28^. P188 is composed of a central hydrophobic chain of polyoxypropylene flanked by two hydrophilic chains of polyoxyethylene, and is currently approved by the Food and Drug Administration as an anti-viscosity agent. The most well documented characteristic of P188 is its ability to repair damaged cell membranes by mechanisms that are not entirely clear. It is believed that P188 inserts into areas of low membrane tension (perhaps areas of membrane damage) due to its amphiphilic and hydrophobic properties^29^. P188 has been shown to reduce infarct size following I/R injury in dogs and pigs when administered acutely^30–32^. Moreover, a 30-min intravenous infusion of high dose P188 (460 mg/kg) was shown to acutely (~ 40 h) improve LV ejection fraction and decrease LV end-systolic volume when administered to rats 8 weeks after acute ligation of the left anterior descending artery^27^. Although we were able to confirm the findings of Martindale et al., which showed that administration of P188 improved recovery of LV function in *Dysf*^*−/−*^ subjected to I/R injury ex vivo (Supplemental Figure S3)^10^, chronic treatment with P188 did not decrease infarct size, nor the extent of adverse LV remodeling following I/R injury in the *Dysf*^*−/−*^ mice. Although the reasons for the discrepancy between the present findings and the prior short term studies are not known, one possibility is that the dose of P188 that we employed was suboptimal for the experimental conditions studied herein. This statement notwithstanding, the dose and dosing strategy used in the present study were based on recommendation by the manufacturer, prior in vivo studies with P188^27,31^, as well as a pharmacokinetic study by the manufacturer that demonstrated the feasibility of the dose that was chosen. A second interesting possibility suggested by our in vitro studies is that P188 was less bioavailable within the infarct zone because of phagocytic uptake by macrophages^33^. While direct correlations between relatively short-term experimental studies in mice and longer-term clinical studies in humans are not appropriate, our data are consistent with the results of the CORE (Collaborative Organization for the RheothRx Evaluation) phase II clinical trial, which did not show a benefit of P188 on the primary end point of mortality, reinfarction or cardiogenic shock in patients with ST-segment myocardial infarction undergoing thrombolytic therapy^34^.

## Conclusions

This study reveals a previously unappreciated role for the importance of membrane sealing and the resolution of inflammation following myocardial injury. The observation that inflammation was sustained within the infarct zone of the hearts of *Dysf*^*−/−*^ mice at a time when inflammation was completely resolved within the infarct zone of WT mice suggests that the inability to reseal membranes in dysferlin-deficient injured cardiac myocytes results in the sustained release of intracellular molecules (e.g. DAMPs) that engage TLR2/TLR4 receptors on cardiac myocytes and immune cells within the infarct zone, leading to the activation of TIRAP-dependent signaling cascades that foster sustained recruitment of inflammatory cells to the infarct zone, resulting in increased cell death, increased infarct size and adverse LV remodeling. While our studies focused on the role of TIRAP dependent TLR2/4 mediated signaling, we cannot exclude an important role for endosomal TLRs or DNA-sensing receptor cyclic GMP–AMP synthase (cGAS) and signaling effector stimulator of interferon genes (STING) in terms of mediating sustained inflammation following I/R injury in *Dysf*^*−/−*^ mouse hearts^35^. Given the increasing recognition that cytosolic proteins (e.g. troponin) are released from cardiac myocytes in non-ischemic settings^36^, the results of the present studies also serve the heuristic purpose of focusing future studies on the role of membrane integrity in the development of the chronic inflammation that occurs in the setting of hemodynamic pressure and volume overload^37–39^, as well as on the need to better understand the molecular pathways that regulate membrane integrity in health and disease.

## Methods

### Study approval

All studies were performed with the approval of the Institutional Animal Care and Use Committee at Washington University School of Medicine. These investigations conform to the *Guide for the Care and Use of Laboratory Animals*, published by the National Institutes of Health.

### Transgenic mouse lines

The dysferlin null mice (B6.129-*Dysf*^*tm1Kcam*^/J[*Dysf*^*−/−*^], Jackson Labs) were maintained on a C57BL/6 background^18^. The *Tirap*^*−/−*^ mice were a gift from Dr. Ruslan Medzhitov (Yale University, New Haven, CT)^40^. Male *Dysf*^*−/−*^ mice were crossed with *Tirap*^*−/−*^ mice to generate *Dysf*^*−/−*^/*Tirap*^*−/−*^ double knockout mice. The *Dysf*^*−/−*^/*Tirap*^*−/−*^ mice were born with the expected Mendelian frequency and had normal life spans. All mice were maintained in a pathogen‐free environment and were fed pellet food and water ad libitum.

### Ischemia reperfusion (I/R) injury in vivo

WT, *Dysf*^*−/−*^, *Tirap*^*−/−*^, and *Dysf*^*−/−*^/*Tirap*^*−/−*^ mice were subjected to ischemia reperfusion (I/R) injury, using a closed-chest ischemia reperfusion model, as described^41,42^. We used 10–12 week old male mice, anesthetized with 1.5% isoflurane, that were subjected to 60 min of closed-chest ischemia, followed by reperfusion for 2–4 weeks. Sham-operated animals underwent the identical procedure with the exception that ischemia was not induced. For experiments using Poloxomer 188 (P188, MAST Therapeutics), mice were given 460 mg/kg P188 via jugular vein infusion immediately following reperfusion. Mice were then given a bolus of 2,000 mg/kg P188 i.p. 6 h later. Daily i.p. injections of 460 mg/kg P188 were then given for 14 days at which time echoes were performed and hearts were harvested for histology. The dose was determined based on the literature^10,26^, and the dosing strategy was based on prior studies by MAST Therapeutics.

### Ischemia reperfusion (I/R) injury ex vivo

Hearts from 10 to 12 week old male WT and *Dysf*^*−/−*^ mice were isolated and perfused at a constant pressure of 70 mmHg with modified Krebs–Henseleit buffer as described previously^8,43^. After a 20-min stabilization period, hearts were subjected to no-flow ischemia (t = 0 min) for 30 min followed by reperfusion (t = 30 min) for up to 60 min (t = 90 min). For experiments with P188, Krebbs Henseleit buffer was prepared with 10 µM P188^10^. KH buffer containing P188 was perfused during baseline and during reperfusion. Functional data were recorded at 1 kHz on a data acquisition system (PowerLab; ADInstruments). LV developed pressure (LVDP) was calculated as the difference between peak systolic pressure and LVEDP, and the resulting LV functional recovery data are expressed as the percentage of LVDP at baseline.

### Echocardiographic studies

#### Image acquisition

Ultrasound examination of the cardiovascular system was performed using a Vevo 2100 Ultrasound System (VisualSonics Inc, Toronto, Ontario, Canada) equipped with a 30 MHz linear-array transducer, as previously described^42^.

#### Imaging protocol

Mice were imaged by echocardiography at baseline and simultaneously during the imposition of closed-chest ischemia, and then at 2 weeks and 4 weeks after I/R injury to evaluate parameters of LV remodeling as described^43^. Noninvasive evaluation of the AAR was performed by assessing the segmental wall motion score index (SWMSI) at the time of reperfusion, as described^43^. Isoflurane (1.5% inhaled via nose cone) was used during ischemia and Avertin (0.005 ml/g) was used for sedation for imaging at 2 and 4 weeks based on previously established methods for infarct quantitation in vivo^42^.

### Evans Blue dye injection

1% Evans Blue dye (Sigma, St. Louis, MO) in PBS was injected i.p. 18 h prior to harvesting hearts. Hearts were harvested, perfused with 10 ml PBS followed by 10 ml Z-fix and allowed to sit in Z-fix overnight before being transferred to 70% ethanol prior to paraffin embedding^43^.

### Histologic analysis

Two weeks after I/R injury mice were euthanized and the hearts were removed and processed, paraffin-embedded, and stained with hematoxylin/eosin and Masson’s trichrome^44^. To estimate the volume fraction (%) of collagen, mid-papillary sections of Masson’s trichrome-stained sections were quantified using color thresholding by ImageJ software (NIH, Bethesda, MD), and data were expressed as the average percentage of the left ventricle stained (n = 5–6 per group). To evaluate myocyte size, sections were deparaffinized, rehydrated, and stained with fluorescent rhodamine-conjugated wheat germ agglutinin at 5ug/ml in 1% BSA, 1X TBS (Vector Labs, Burlingame, CA). Fluorescence was visualized using a Zeiss confocal microscope, and digital images were analyzed and measured with Zeiss Axiovision software^45^.

We enumerated the CD68+ and Ly6G+ cells in the heart via 10X tile scanning of the entire heart at the mid-papillary level. Hearts were fixed in 4% PFA overnight and then transferred to 30% sucrose for 24 h prior to embedding in OCT and flash freezing. Prior to immunostaining, frozen sections were washed briefly in PBS and then blocked in 3% BSA/5% horse serum. Sections were then incubated with antibodies to CD68 (MCA1957, Biorad) or Ly6G (551459, BD) followed by Alexa 555 conjugated secondary antibody incubation (A224134, Fisher). Fluorescence was visualized using a Zeiss confocal microscope. Images (~ 70–130 per section) were stitched together using the Axiovision software to form whole mid-papillary section images (Supplemental Figure S5) to visualize the infarct zone. CD68+ and Ly6G+ cells were then manually counted in each tile comprising the infarct zone to quantitate.

### Bone marrow transplantation

We established reciprocal *Dysf*^*−/−*^ and WT chimeric mice after lethal irradiation and bone marrow reconstitution. Chimeric mice were generated as described^46^. Briefly, 4-week-old WT and *Dysf*^*−/−*^ mice were irradiated with 1,000 Rads and reconstituted via intravenous injection with congenic 5 × 10^6^ bone marrow cells isolated from the tibias and femurs of 4 to 5-week-old WT mice to generate WT → WT and *Dysf*^*−/−*^ → WT chimeras. Mice were allowed 8 weeks to reconstitute the bone marrow before use for experiments.

### Peritoneal macrophages

Intraperitoneal macrophages (ipMACs) were isolated as previously described^47^. Cultures of ipMACs were stimulated with diluent, P188 (1.5 mg/ml final concentration), necrotic myocardial cell extracts (10 µg/ml; positive control)^5^, and lipopolysaccharide (100 ng/ml; positive control [Sigma, St. Louis, MO]) for 24 h. IL-1β was measured in stimulated cell culture supernatants using an IL-1β ELISA kit (R&D kit Cat. #MLB00C) according to the manufacturer’s instructions. The phagocytic capacity of stimulated cultures of ipMACs was assessed by measuring phagocytosis of 0.5um Fluoresbrite yellow-green fluorescent polystyrene microspheres (Polysciences Inc., Warrington, PA). For these experiments ipMACs were plated on coverslips and incubated with microspheres for 2 h, at a ratio of 200 microspheres per cell, and then cultures were fixed with 4% paraformaldehyde. Cells were then incubated with anti CD68 antibody (#MCA1957, Biorad, Hercules, CA), followed by incubation with goat anti-rat 594 secondary antibody (#A11007, Thermofisher, Waltham, MA) and mounted with DAPI containing Vectashield mounting media (Vector Laboratories, Burlingame, CA). Fluorescence was imaged via confocal at 20× and quantitated. CD68 positive cells that had internalized more than 5 microspheres were considered positive for phagocytosis. In separate experiments, macrophages were incubated with FITC-labeled P188 (1.5 mg/ml; prepared by the Center for Drug Discovery, Washington University and Aris Pharma, Levittown, PA) for 18 h and the ipMACs imaged using fluoresence confocal microscopy.

### Statistical analysis

Data are expressed as means ± SEM. One-way ANOVA with Bonferroni post-hoc analysis (Graphpad Prism) was used to test for differences in EBD uptake, Trichrome staining, cell area, CD68 and Ly6G quantification. For repeated measures analysis, (2-D echocardiography and Langendorff perfusion data), mixed models methodology or 2- way repeated measures ANOVA were used. For the mixed model, group, time and the interaction between group and time were all entered into the model. Time was treated as a categorical variable. The interaction was evaluated to determine if the relationship between group and echo parameter varied with respect to time. Based on the Akaike Information Criteria (AIC) and Shwarz Information Critieria (BIC), an unstructured variance–covariance matrix was used to model the repeated measurements. Group means were based on model results and differences between groups were determined overall and within each time point. All pair-wise comparisons were considered. Test results were adjusted based on Sidak’s method to control the overall type I error rate. A value of *p* < 0.05 was considered statistically significant^43^.

## Supplementary information

Supplementary Information 1.

## Data Availability

The authors agree to make all materials, data and associated protocols available to readers promptly without undue qualifications in material transfer agreements.
