# Supplementary material for: Ischemia reperfusion injury provokes adverse left ventricular remodeling in dysferlin-deficient hearts through a pathway that involves TIRAP dependent signaling
Source: Sci Rep. 2020 Aug 24;10:14129. doi: 10.1038/s41598-020-71079-7 (PMC7445276; doi:10.1038/s41598-020-71079-7)
Supplement: Supplementary file 1 — Supplementary Information 1. [file 41598_2020_71079_MOESM1_ESM.pdf]

Supplementary Information

Ischemia Reperfusion Injury Provokes Adverse Left Ventricular Remodeling in Dysferlin-Deficient Hearts Through a Pathway That Involves TIRAP Dependent Signaling

Sarah Evans, PhD[1]

Carla J. Weinheimer, MS[1]

Attila Kovacs, MD[1]

Jesse W. Williams, PhD[2]

Gwendalyn J. Randolph, PhD[2]

Wenlong Jiang, MD[1]

Philip M. Barger, MD[1]

Douglas L. Mann, MD[1]

[1]Center for Cardiovascular Research, Cardiovascular Division
Washington University School of Medicine, St. Louis, MO
[2]Department of Pathology and Immunology
Washington University School of Medicine, St. Louis, MO

Address for Correspondence:
Douglas L. Mann, MD
Division of Cardiology
660 S. Euclid Ave, Campus Box 8086

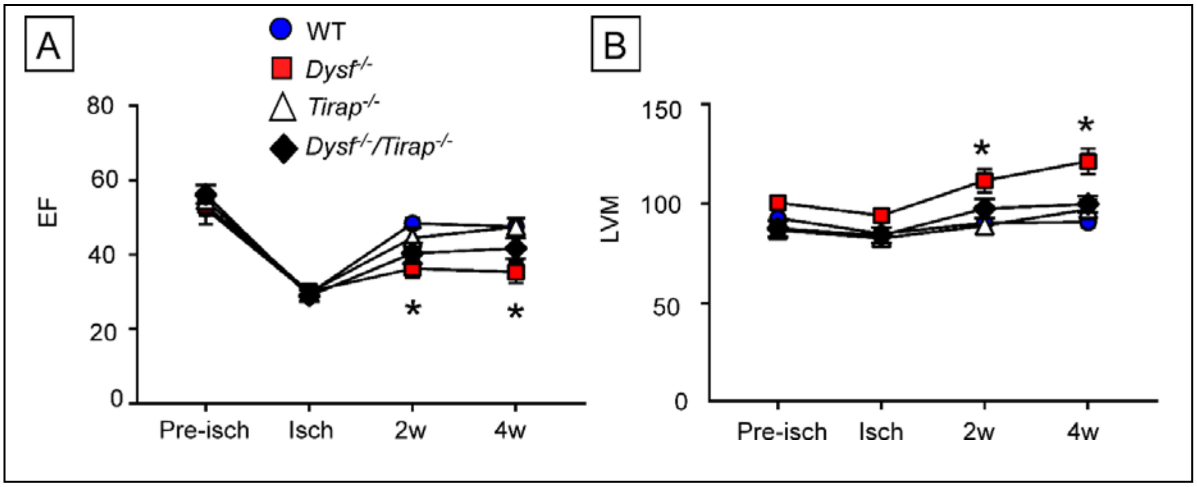

Supplemental Figure S1. Effect of ischemia reperfusion (I/R) injury in WT, *Dysf*$^{-/-}$, *Tirap*$^{-/-}$ and *Dysf*$^{-/-}$/*Tirap*$^{-/-}$ mouse hearts. WT (n=11), *Dysf*$^{-/-}$ (n=17), *Tirap*$^{-/-}$ (n=9), *Dysf*$^{-/-}$/*Tirap*$^{-/-}$ (n=9) mice underwent closed chest ischemia (60 min) followed by 2 to 4 weeks of reperfusion. Mice were imaged by 2-D echocardiography at baseline (pre-ischemia), during the imposition of ischemia, and at 2 and 4 weeks. **(A)** Left ventricular ejection fraction (EF) **(B)** Left ventricular mass (LVM). Data are presented as mean±SEM. (*p<0.05 compared to WT)

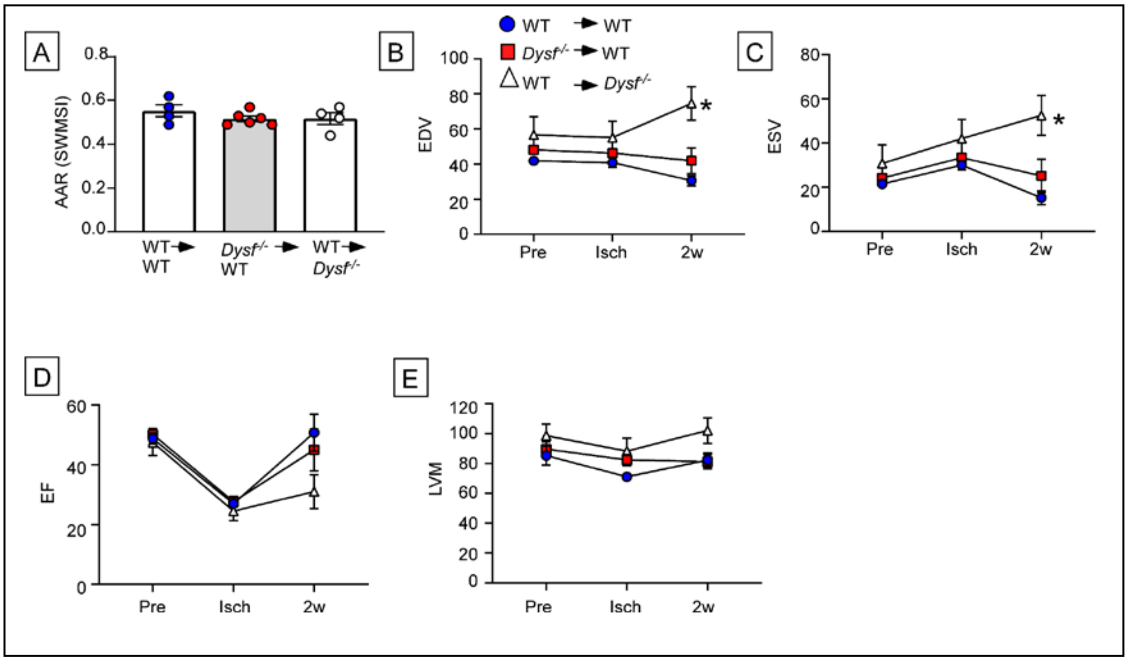

Supplemental Figure S2. Effect of I/R injury in chimeric WT mice. WT mice that underwent bone marrow reconstitution with WT bone marrow (WT → WT) or *Dysf*$^{-/-}$ bone marrow (*Dysf*$^{-/-}$ → WT) and *Dysf*$^{-/-}$mice that underwent bone marrow reconstitution with WT bone marrow (WT → *Dysf*$^{-/-}$) were subjected to closed chest ischemia (60 min) followed by 2 weeks of reperfusion. Mice were imaged by 2-D echocardiography at baseline (pre-ischemia), during ischemia, and at 2 weeks (n= 4-6/group). **(A)** Area-at-risk (AAR [SWMSI]), **(B)** Left ventricular end-diastolic volume (EDV), **(C)** Left ventricular end-systolic volume (ESV), **(D)** Left ventricular ejection fraction (EF), **(E)** Left ventricular mass (LVM), Data are presented as mean ± SEM. (* $p < 0.05$ compared to WT→WT)

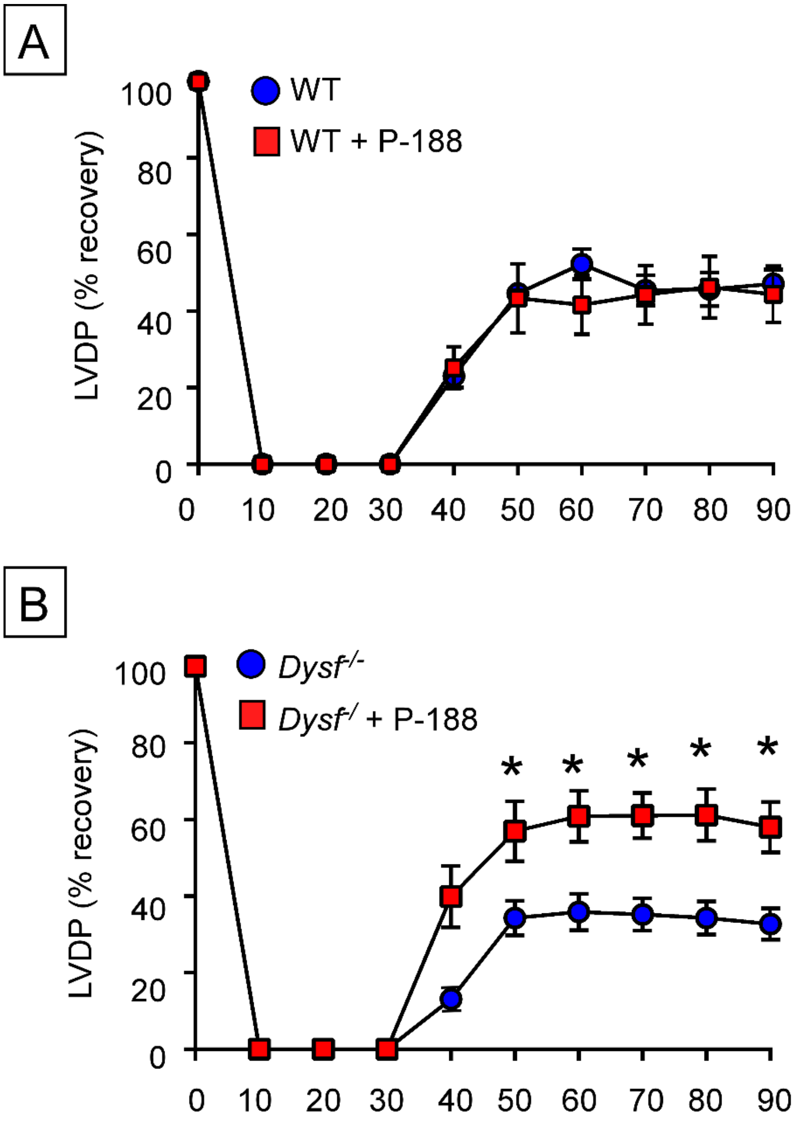

Supplemental Figure S3. Effect of Poloxamer 188 (P188) on recovery of LV function in WT and *Dysf*$^{-/-}$ mice following I/R injury ex vivo. Hearts from WT and *Dysf*$^{-/-}$ mice were subjected to 30 min ischemia followed by reperfusion for 60 minutes ex vivo using a Langendorff apparatus. Hearts were perfused with P188 (10 µM) or diluent during baseline and during reperfusion. Recovery of LV function was determined by measuring the percent of LV developed pressure (LVDP) after I/R injury. **(A)** Effect of P188 on LVDP in WT mice following I/R injury (n=5-8 per group), **(B)** Effect of P188 on LVDP in *Dysf*$^{-/-}$ mice following I/R injury (n=4-7 per group). Data are presented as mean±SEM. (*p<0.05 compared to WT).

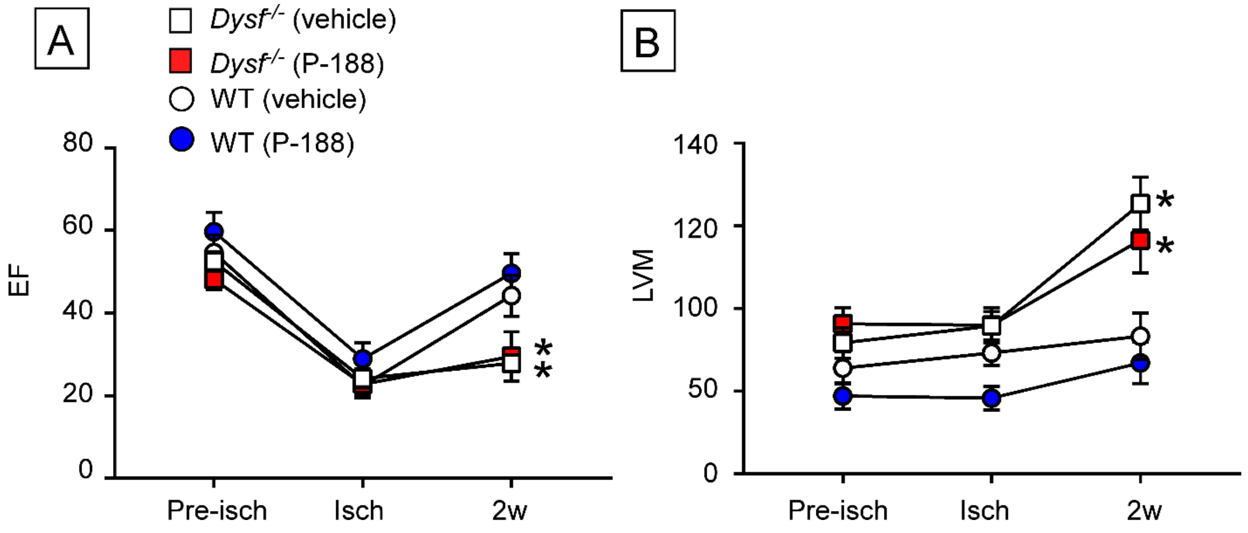

Supplemental Figure S4. Effect of Poloxamer 188 (P188) on left ventricular ejection fraction and left ventricular mass in WT and *Dysf$^{-/-}$* mice following I/R injury. WT and *Dysf$^{-/-}$* mice, subjected to closed chest ischemia (60 min) or a sham procedure followed by 2 weeks of reperfusion, were treated with P188 or an equal volume of diluent immediately following reperfusion, 6h later and then daily for 14 days. Mice were imaged by 2-D echocardiography at baseline (pre-ischemia), during ischemia and at 2 weeks after reperfusion (n = 5-6/group). **(A)** Left ventricular ejection fraction (EF), **(B)** Left ventricular mass (LVM). Data are presented as mean±SEM. (*$p<0.05$ compared to WT)

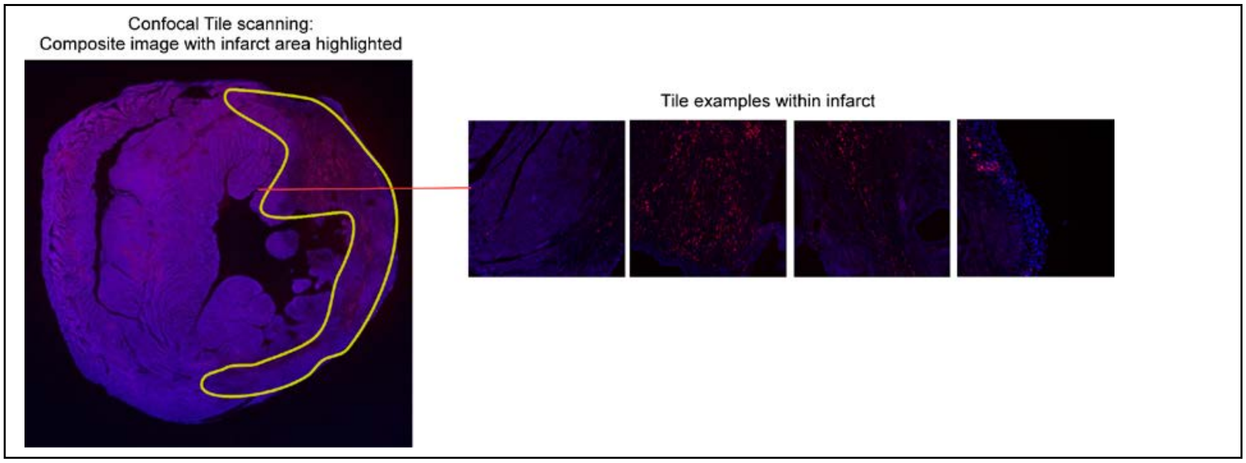

Supplemental Figure S5. Confocal tile scanning for enumerating CD68+ and Ly6G+ cells within the infarct zone. 10X tile scanning was performed at the mid-papillary level using confocal microscopy. Images (~70-130 per section) were stitched together using the Axiovision software to form whole mid-papillary section images. Individual sequential tiles are shown to the right of the composite image to demonstrate how CD68+ and Ly6G+ cells were enumerated within the infarct zone.
